# Use of Collodion in Exposure of Nerves

**Published:** 1859-09

**Authors:** Abr. Robertson

**Affiliations:** Wheeling, Va.


### DENTAL REGISTER OF THE WEST.

Vol. XIII.] SEPTEMBER, 1859. [No. 3.

### Original Essays and Communications.

### USE OF COLLODION IN EXPOSURE OF NERVES.

BY ABR. ROBERTSON.

At the meeting of the American Society of Dental Surgeons (that was) which was held in Philadelphia eight years ago, I communicated verbally, but very briefly, a method which I had then been using, under certain circumstances, in the treatment of exposed nerves, for about two years; a very meagre report of which was published at the time, in some of the journals, among the transactions of the meeting.

An experience now of about ten years in the same course of treatment, and with almost uniform success, induces me to speak of it, and to recommend it to the numerous readers of the Register with great confidence.

I have intimated above that it is only under certain, and I will now add, favorable circumstances that I regard the proposed treatment as applicable. For I believe that whenever the nerve is exposed, and has become inflamed, and especially if it is so much inflamed as to be painful, it had better be destroyed at once; and before reading your "Thoughts on fang-filling" in the last number of the Register, I should have said—their canals thoroughly cleaned out and filled to their points. But now, for myself, I shall try the *tanning* process and test your reasoning, and which certainly seems plausible. And I will further digress enough to say that I have frequently succeeded better, and to my surprise too, sometimes, in the filling of roots in very bad cases where I have deemed it necessary to adopt a preparative course of treatment similar to what you recommend, than I have in many other cases that seemed far more favorable and where I had not deemed it necessary thus to treat them. In the bad cases I have frequently healed old fistulous abscesses; in the favorable cases I have sometimes caused them: I am therefore inclined to think your theory correct. But my present purpose is not to speak of cases where the nerves have been, or ought to be, destroyed. It is simply to call attention to a method by which many nerves may be preserved in health, that would otherwise be destroyed—and in destroying which, as sometimes happens, one of the teeth to which they belong, too might be destroyed.

Whenever, for example, in preparing a cavity for filling I accidentally, or necessarily expose a nerve, if there are no appearances of inflammation in it—if it have not already ached, I proceed to finish my preparation as if the nerve had not been exposed. If in exposing the nerve I have wounded the membranes so as to cause bleeding, I wait until the bleeding has ceased; I then wash out the cavity and dry it thoroughly with cotton and tissue paper as in any other case. I then apply a drop of collodion over the exposed nerve, wait till the ether has evaporated from it, and then proceed to fill without any further especial care except to adapt my foil as smoothly as possible to the part where the nerve was wounded.

If the cavity is so situated that I can not otherwise apply the collodion, I take a very small piece of tissue paper on the point of an instrument, dip it in the collodion and then spread it as smoothly as possible over the point to be protected, wait the evaporation of the ether as before directed, and then fill.

Although I am fully of the opinion that Dr. Arthur's method of leaving the *softened* bone to protect the nerves of such teeth as would be exposed by its removal, taking care to remove all in the vicinity of the margins of the cavity so to make a firm foundation for the filling, and to make it impervious to fluids, although it has been pretty severely criticised is worthy of consideration, and in many cases, at least, may be the best treatment; and that therefore great care should always be taken to avoid unnecessarily exposing the nerves of teeth; still accidents will sometimes happen, and the exposing of a nerve is sometimes unavoidable, and in such cases this method in my hands has succeeded far better than any other I have ever tried or seen tried, and by it I am sure I have saved the nerves of a great many teeth that otherwise would have been lost.

The old fashioned methods of capping, with either lead or gold never succeeded in my hands; and I have but rarely if ever known it to succeed in the hands of others.

I have been familiar with the use, and have observed the effects of nitric, or of nitro-muriatic acids in such cases, about the same length of time that I have used this. Sometimes I have seen it succeed, and in many cases I have seen it fail.

I was familiar, too, with the famous "risodontrypy" or "Hullihen operation" about a year before Dr. Hullihen caused it to be publicly promulgated in the report made by the late Dr. C. O. Cone, at the meeting of "the late" American Society of Dental Surgeons at Newport, R. I., in August, 1852; the Doctor having himself written me a description of his operation with a request that I should try it and tell him of my success. For his sake I tried it a great many times, having first however expressed to him my entire want of confidence in an operation which *seemed* so entirely unphilosophical, and therefore so unlikely to succeed.

With that operation I sometimes succeeded, but most frequently failed. And of many of the operations where at first I thought I had succeeded, "time told a different," though not a "flattering tale." Such too, I am satisfied, was the result of the operation in Dr. Hullihen's own hands. I know, at least, by an observation of about two years now, in the same field where he labored, that a great many of his operations of that kind were failures. But in a pretty extensive use of the collodion for about ten years, I can hardly call to mind a failure—none where anything else ought reasonably to have been expected.

The collodion by being liquid must of course adapt itself perfectly to the wounded surfaces; and as it is very elastic when dry it produces no irritation, or far less at any rate than any unyielding substance could, even if it could fit as perfectly. By its contraction in the evaporation of the ether it most effectually prevents further bleeding during the operation of filling—a matter of first-rate importance—and it also to some extent insulates the nerve from the effects of changes of temperature, it being a less perfect conductor of caloric than the metal.

Wheeling, Va., July 21, 1859.

